# Renal cell carcinoma in children: case report and literature review

**DOI:** 10.11604/pamj.2015.20.84.5791

**Published:** 2015-01-29

**Authors:** Aissa Abdellah, Kadiri Selma, Marnouche Elamin, Touil Asmae, Rouas Lamia, Malihy Abderrahmane, El Majjaoui Sanaa, Elkacemi Hanan, Kebdani Tayeb, Benjaafar Noureddine

**Affiliations:** 1 Department of Radiation Oncology, National Institute of Oncology, Ibn Sina University Hospital, Mohamed 5 Souissi University, Rabat, Morocco; 2 Department of Pathology, pediatrics hospital, Ibn Sina University Hospital, Mohamed 5 Souissi University, Rabat, Morocco

**Keywords:** Renal cell carcinoma, children, surgical resection

## Abstract

Renal cell carcinoma is infrequent in children; consequently it is important to communicate its diagnosis and follow up. The behaviour of this type of tumor is better characterized in adults and in this setting the treatment of choice is surgical resection. However, the place of chemo- and radiotherapy has not been well defined. Here, we present a 9-year-old boy with renal cell carcinoma demonstrating only hematuria without any pathological physical examination findings. The mass was described by abdominal ultrasonography and computed tomography in the left kidney. After the left nephroureterectomy, the patient was given no adjuvant therapy.

## Introduction

Renal cell carcinoma (RCC) usually occurs in the age range of 50 to 70 years. It is extremely rare in children, similar to Wilms’ tumor. The incidence of RCC tumor in childhood is estimated to be from 1.8% to 6.3% of all malignant renal tumors [1]. The biologic behavior and the prognostic factors of RCC are not well-known but may resemble that in adults. So far, no treatment protocols have been defined for children with RCC. Surgery is the mainstay of treatment and results in cure when the tumor is localized and completely resected. The importance of radiotherapy and immunotherapy is not clear and different chemotherapy regimens showed only minimal activity when tested in clinical trials [1]. Here, we present an 9-year-old patient with RCC presenting with hematuria.

## Patient and observation

**Figure 1:**
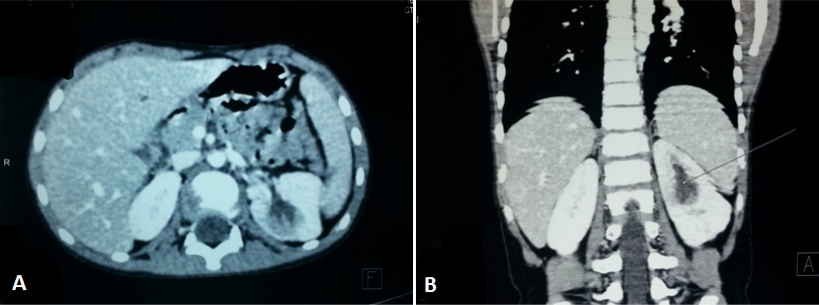
Computed tomographic images showing a heterogeneous left renal mass measuring 40/36/30 mm: A) scannographic axial section; B) frontal reconstruction

**Figure 2:**
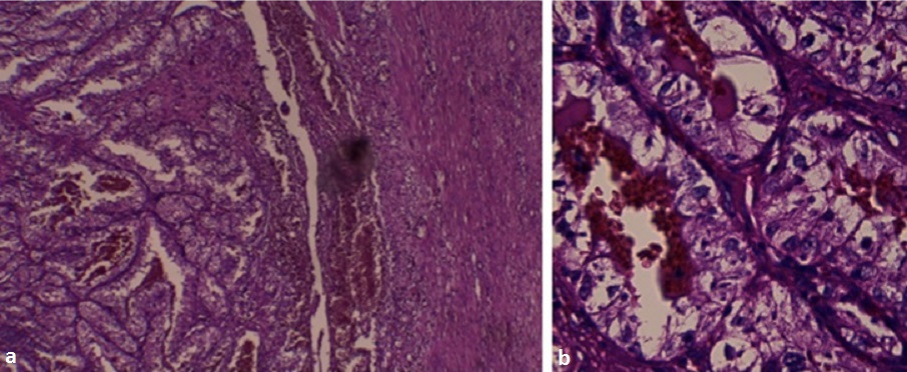
Histologic appearance: the tumor cells have clear cytoplasm and are arranged in nests with intervening blood vessels (GX40 (a) and GX200(b))

An 9-year-old boy was referred to our institution for eventual adjuvant radiotherapy of renal cell carcinoma in the right kidney. Gross hematuria was the outstanding feature initially. Ultrasound and abdominal CT did not determine between a tumor or infectious origin (Figure 1). Patient underwent transabdominal nephrectomy with regional lymphadenectomy. The tumor has 8 cm in diameter, the renal capsule was intact, and there was no tumor thrombus in the renal vein or inferior vena cava. Histopathology revealed RCC with granular cytoplasm and pleomorphic nuclei (Figure 2). Hilar lymph nodes were negative, indicating he was a stage I RCC requiring no further therapy. The boy recovered well and was disease free during the follow-up period of 18 months.

## Discussion

Renal cell carcinoma represents 2% of malignant tumors in adults and is the third most frequent tumor of the urinary tract after prostate and bladder tumors. On the other hand, in pediatric ages, only 2% to 3% of malignant renal tumors are proved to be RCC [2, 3]. The incidence of RCC increases with age. According to the survey of Japanese Society of Paediatric Surgeons, RCC accounted for 1.4% of all renal tumors in patients younger than 4 years, 15.2% in patients aged 5 to 9 years, and 52.6% in patients aged 10 to 15 years [4]. It is more frequent in older age than 5 years, reaching the incidence of Wilms′ tumor in the second decade of life. Whereas the peak of incidence in Wilms′ tumor occurs around 3 years of age, RCC presents between 9 and 15 years of age [5, 6].

Generally, there is no sex predominance for this renal tumour type in children in the literature [2] unlike in adults, in which the tumour predominates in males. The most common form of presentation of RCC in children is macroscopic hematuria and abdominal or flank pain. Other less frequent symptoms are palpable abdominal mass, anaemia, and fever [2, 5]. Its presentation as an incidental finding in children is less common than in adults. RCC is diagnosed as a solid mass with US and CT. However, it is generally difficult to differentiate it from Wilms’ tumour before surgery in children. Calcification or high-density areas on CT have been reported in 14% to 28% of RCCs in children [6, 7]. RCC is a malignant tumour arising from epithelial cells of the renal tubules. RCCs comprising a heterogeneous group of tumours are classified with 5 subtypes: clear-cell RCCs, papillary RCCs, chromophobe RCCs, collecting duct carcinomas, and unclassified RCCs. This classification reflects the location within the nephron in which the tumors originate [8].

Surgical resection is the mainstay of therapy for RCC given its intrinsic resistance to chemotherapy and radiation therapy. Debate continues about the role of lymph node dissection at the time of nephrectomy. Geller and Dome [9] combine their own institutional experience with a review of the literature. They noted 72.4% disease free survival in patients with nodal disease (N + ). Among N+ patients who underwent adjuvant chemotherapy or radiation therapy, no improvement in disease-free or overall survival was noted. Geller and Dome conclude that lymphadenectomy in the absence of clinical or radiographic suspicion for nodal involvement confers no benefit. However, the preoperative diagnosis of tumour in children is difficult and the effects of chemotherapy, including immunotherapy, are unclear. Postoperatively, adjuvant radiotherapy and chemotherapy have been used in patients with higher-grade tumours. Although immunotherapies with interferon or interleukin for the treatment of advanced cases have been reported, the beneficial effects of these treatments are uncertain in children because of the lack of any prospective randomized studies [10]. The role of new agents such as tyrosine kinase inhibitors is completely undefined in paediatric RCC. Given the overexpression of MET tyrosine kinase in ASPL/TFE3 translocation tumors, Geller et al [11] speculate that the MET inhibitors currently in trials may be helpful in translocation RCC.

The review of literature reveals the overall survival rate of paediatric RCC to be around 63%, with survival rates for stages I to IV at 92.4%, 84.6%, 72.7%, and 13.9%, respectively [6]. Patient age, tumour size, histological pattern, and vascular invasion have all been reported to be the predictors of outcome. Most recurrences and deaths usually occur within the first 2 years after diagnosis, although late recurrences are frequent [1]. So a strict long-term followup is required.

## Conclusion

Although RCC is rare in children, clinical suspicion of this disease in children older than 5 years with renal masses is very important since the diagnostic and therapeutic approach differs from that for Wilms’ tumor. Radical nephrectomy associated with regional lymphadenectomy is the best treatment for RCC in childhood. The disease appears to have a less aggressive behavior in children. The main prognostic factors seem to be staging and complete resection.
